# Antibacterial and antibiofilm activities of ZIF-67

**DOI:** 10.1038/s41429-023-00637-8

**Published:** 2023-06-19

**Authors:** Ramses Gallegos-Monterrosa, Rodrigo Orozco Mendiola, Yoselin Nuñez, Constance Auvynet, Kesarla Mohan Kumar, Bin Tang, Leonardo I. Ruiz-Ortega, Víctor H. Bustamante

**Affiliations:** 1 Departamento de Microbiología Molecular, Instituto de Biotecnología, Universidad Nacional Autónoma de México, C.P. 62210 Cuernavaca, Morelos México; 2 Instituto de Ciencias Físicas, Universidad Nacional Autónoma de México, C.P. 62210 Cuernavaca, Morelos México; 3 Departamento de Medicina Molecular y Bioprocesos, Instituto de Biotecnología, Universidad Nacional Autónoma de México, C.P. 62210 Cuernavaca, Morelos México; 4 Department of Biomedical Engineering, Southern University of Science and Technology, 1088 Xueyuan Avenue, Shenzhen, 518055 PR China; 5 Department of Biological Sciences, Columbia University, New York, NY 10027 USA

**Keywords:** Nanomedicine, Antibiotics

## Abstract

Currently, antibiotic-resistant bacteria represent a serious threat to public health worldwide. Biofilm formation potentiates both virulence and antibiotic resistance of bacteria. Therefore, the discovery of new antibacterial and antibiofilm compounds is an issue of paramount importance to combat and prevent hard-to-treat bacterial infections. Zeolitic-imidazolate-frameworks (ZIFs) are metallo-organic compounds known to have various interesting chemical and biological applications, including antibacterial properties. In this study, we synthesized ZIF-67 nanoparticles, formed by imidazolate anions and cobalt cations, and found that they inhibit the growth of *Acinetobacter baumannii*, *Pseudomonas aeruginosa*, and *Staphylococcus aureus*. Sub-inhibitory concentrations of ZIF-67 were also able to significantly reduce the biomass of pre-established biofilms of these pathogenic bacteria. On the other hand, the ZIF-67 nanoparticles had null or low cytotoxicity in mammalian cells at those concentrations showing antibacterial or antibiofilm activities. Thus, our results reveal the potential of ZIF-67 nanoparticles to be used against pathogenic bacteria.

## Introduction

Infections triggered by antibiotic resistant bacteria are considered a major global health threat by the World Health Organization (WHO). It was estimated that in 2019 1.27 million deaths were attributable to infections caused by antibiotic resistant bacteria [1]. Without the development and implementation of new approaches to treat bacterial disease, the number of deaths is projected to reach 10 million people per year by 2050, which may represent up to US$ 100 trillion in health care cost [2, 3]. The WHO has published a list of pathogenic bacteria for which new antibiotics and other antibacterial compounds are urgently needed, including *Acinetobacter baumannii, Pseudomonas aeruginosa*, and *Staphylococcus aureus* [4]. These bacteria have emerged as important causative agents of hospital-acquired infections, especially in patients with weakened immune systems such as the elderly and patients with diverse chronic or terminal diseases. Clinical strains of *A. baumannii*, *P. aeruginosa* and *S. aureus* commonly show multi- or even pan-antimicrobial resistance, thus making their infections hard to treat. Furthermore, these bacterial species, among many others, are able to form biofilms [5–7]. Bacterial biofilms are communities characterized by the presence of a protective matrix of biopolymers that encases and protects the cells living within it, providing them with a remarkable resistance to external insults that allows them to persist in hospital environments [8]. Nowadays, pathogenic bacterial biofilms are recognized as a serious and costly problem for human health: their resistance to environmental threats makes them hard to eliminate, and they can thus establish chronic infections. It is estimated that cells living in a biofilm can be up to 1000 times more resistant to antibiotics than their planktonic counterparts [9]. Bacterial biofilms can be formed on a variety of medical equipment and implants such as catheters, joint prostheses and pacemakers among others, medical devices with an associated biofilm infection can suffer a loss in their functionality. Since established biofilms are difficult to eliminate, these medical devices often have to be replaced, which causes extra costs and risks to the patients [10, 11]. As a consequence of this problematic, a new scientific field of antibiofilm materials research has developed, where diverse materials and strategies are being investigated to prevent the establishment of bacterial biofilms on medical devices [11, 12]. Additionally, there is a recognized need for compounds that can eliminate bacterial biofilms once they are established.

Nanomaterials have been investigated in recent years as alternative antibacterial compounds, in particular, metal and carbon-based complexes have shown the capacity to eliminate bacteria via the release of metal ions, generation of reactive oxygen species, and other mechanisms [13, 14]. However, the potential toxicity of these materials to the patient and environment, mainly due to released metal ions, must be carefully considered as well [15]. Metal organic frameworks (MOFs) are a type of nanomaterials that exhibit different properties, including antibacterial activity. MOFs consist of microporous hybrid organic-inorganic crystalline structures, composed of transition metal nodes and polyfunctional organic linkers as subunits, which can self-assemble into 3D structures and can function as molecular carriers or delivery vehicles for drugs and other compounds [16]. Transition metal-based MOFs have also shown antibacterial activity through the release of Co, Cu, Zn, and Mn ions that affect bacterial cell walls [17, 18]. Zeolitic imidazolate frameworks (ZIFs) are a class of 3D-structured MOFs that exhibit a zeolite type structure [19]. Imidazole derivative compounds are known to have antibacterial, antifungal and antiviral activities [20, 21]. However, information regarding the antibacterial activity and the mechanism of action of ZIFs is scant. Zeolitic imidazolate framework-67 (ZIF-67), formed by bridging 2-methylimidazolate anions and cobalt cations, is known to have various interesting chemical applications such as CO_2_ reduction [22] and water splitting [23]. Recently, ZIF-67 was demonstrated to accelerate healing of infected diabetic chronic wounds [24], and to show antibacterial activity against a laboratory *Escherichia coli* strain when used as ZIF-67/alginate fibers [25].

Here, we synthesized ZIF-67 nanoparticles and analyzed their antibacterial and antibiofilm activities. Interestingly, our ZIF-67 nanoparticles exhibited growth-inhibitory and antibiofilm activities against the pathogenic bacteria *A. baumannii, P. aeruginosa* and *S. aureus* at concentrations that show null or low toxicity against tested eukaryotic cell types.

## Materials and methods

### Synthesis of ZIF-67

ZIF-67 was synthesized following a previously reported procedure [26]. Briefly, 0.45 g of cobalt nitrate hexahydrate were added to 3 ml of double distilled water (solution A) and 5.5 g of 2-methylimidazole were added to 20 ml of double-distilled water (solution B). Solutions A and B were mixed and stirred at room temperature for 6 h and then the purple precipitate was collected by centrifugation. The precipitate was dried in an oven at 80 °C overnight, thus obtaining ZIF-67 powder.

### Characterization of ZIF-67

X-Ray diffraction pattern of synthetized ZIF-67 nanoparticles was recorded on a Burker D8 Advanced eco equipment between 5° and 50° with a step size of 0.02°. Morphological and elemental analysis were obtained using Scanning Electron Microscope JEOL JSM-IT 500 coupled with Energy Dispersive Spectroscopy (EDS).

### Bacterial strains and growth media

Bacterial strains used in this study are listed in Table 1. Bacteria were grown in Mueller-Hinton broth (MH, Difco; 2 g beef extract, 17.5 g casein acid digest, and 1.5 g soluble starch, in 1 liter distilled water) at 37 °C with shaking at 200 r.p.m., unless otherwise stated. Medium was supplemented with Bacto agar (15 g l^−1^) when solid plates were required. When needed, antibiotics were used at the following concentrations: 20 μg ml^−1^ gentamycin, 30 μg ml^−1^ chloramphenicol, and 12 μg ml^−1^ tetracycline.

**Table 1:** Bacterial strains used in this study

Bacteria	Description	Source
*Acinetobacter baumannii* ATCC 17978	Reference strain; Amp^R^	ATCC
*Staphylococcus aureus* ATCC 29213	Reference strain	ATCC
*Pseudomonas aeruginosa* PA01	Reference clinical strain; Cm^R^, Tet^R^	[27]
*Pseudomonas aeruginosa* 27853	Reference strain	ATCC

Antibiotics: ampicillin (Amp), chloramphenicol (Cm), and tetracycline (Tet)

*ATCC* American Type Culture Collection

### Bacterial growth inhibition on agar plates

Bacteria were grown in MH broth up to an optical density at 600 nm (O.D._600nm_) of 0.6. Then, a sample of the culture was diluted in fresh MH broth up to O.D._600nm_ 0.05. Subsequently, 1 ml of the diluted bacterial suspension was evenly spread on MH agar plates. After an incubation of 5 min, the excess liquid was discarded, and the inoculated plates were dried in a laminar flow hood for 10 min. Then, 10 μl of ZIF-67 aqueous solution at 1, 5, 10, or 50 mg ml^−1^ were placed on the inoculated plates. As a positive control for bacterial growth inhibition, 1 μl of antibiotic gentamycin (25 mg ml^−1^) was placed in the center of the plate. The plates were incubated at 37 °C overnight. The presence of an inhibition halo was considered as positive for bacterial growth inhibition.

### Bacterial growth inhibition in liquid medium

Bacteria were grown in MH broth up to an O.D._600nm_ of 0.6. After this, a sample of the cultures was diluted in fresh MH broth up to an O.D._600nm_ of 0.05. Then, 180 μl of the diluted bacterial suspensions were placed in the wells of a 96-well microplate. Immediately, 20 μl of ZIF-67 aqueous solution at 6, 7, 8, or 8.5 mg ml^−1^ were added for each bacterial strain tested. As a growth inhibition control, 20 μl of MH broth containing antibiotic chloramphenicol (12 μg ml^−1^) were added instead of the ZIF-67 solutions, whereas as a positive control for growth, 20 μl of MH broth alone were added. 2-methylimidazole and cobalt nitrate hexahydrate were also assessed at equimolar amounts to those for ZIF-67. Growth kinetics of bacteria were obtained by incubating the inoculated plates at 37 °C and 237 r.p.m for 24 h and measuring absorbance at 600 nm every 30 min, in a microplate reader Epoch2 (BioTek).

Minimum inhibitory concentrations, defined as the lowest concentration that inhibits visible bacterial growth [27], were determined as the concentration that completely inhibited growth (no change in O.D._600nm_) during our growth kinetics assays.

### Bacterial kill rates

Bacteria were grown in MH broth up to an O.D._600nm_ of 0.6. Afterwards the cells from 3 ml of these cultures were pelleted with centrifugation at 10,000 × *g* for 3 min. The supernatant was discarded and the cells were softly resuspended in 1 ml of sterile phosphate-buffered saline (PBS; 1.8 mM NaH_2_PO_4_, 10 mM Na_2_HPO_4_, 2.7 mM KCl, and 137 mM NaCl, in distilled water, pH 7.4). This washing step was repeated three times to eliminate all leftover MH medium. Then, the bacterial suspension was adjusted to O.D._600nm_ of 0.1 in a volume of 2 mL using sterile PBS. 450 μl of the diluted bacterial suspension were mixed with 50 μl of sterile PBS, ZIF-67 at 8 500 μg ml^−1^, or chloramphenicol at 120 μg ml^−1^. The samples were incubated at 37 °C and 200 rpm for 4 h in 1.5 ml microcentrifuge tubes. At timepoints 0 min, 30 min, and 1, 2, and 4 h, 10 μl of the samples were used to prepare 1:10 serial dilutions using PBS. Then, 5 μl drops of each sample and their corresponding dilutions were placed on MH agar plates, and the plates were incubated at 37 °C for 18 h. Finally, the plates were photographed to examine the bacterial kill rates.

### Biofilm eradication

Bacteria were grown up to an O.D._600nm_ of 0.6 in 2×SG broth containing 16 g nutrient broth (Difco), 2 g KCl, 0.5 g MgSO_4_·7H_2_O, 1 g glucose, 1 mM Ca(NO_3_)_2_, 1 mM MnCl_2_·4H_2_O and 1 µM FeSO_4_ per liter distilled water [28]. After this, the bacterial cultures were diluted in fresh 2×SG broth up to O.D._600nm_ 0.05. Subsequently, 200 μl of the diluted bacterial suspensions were placed in the wells of a 96-well microplate, which was then incubated at 37 °C for 24 h without agitation to permit biofilm formation. The planktonic cells were then carefully removed from the wells of the microplate via aspiration with a micropipette and the attached cells were softly washed 3 times with 200 μl of PBS. Afterwards, 200 μl of 2×SG broth containing ZIF-67 at 0, 100, 500, or 1000 μg ml^−1^ were added into the wells. Antibiotics vancomycin (2 and 10 μg ml^−1^), tetracycline (4 and 12 μg ml^−1^), gentamicin (2 and 10 μg ml^−1^), and chloramphenicol (16 μg ml^−1^) were also assessed. The microplate was incubated at 37 °C for additional 24 h without agitation. Then, the amount of biofilm biomass that remained attached to the microplate wells was measured by crystal violet staining as described previously [29]. Absorbance at 570 nm was determined in a microplate reader Epoch2 (BioTek).

### Ethics declaration

Human erythrocytes were obtained from healthy donors. Mouse erythrocytes were obtained from C57BL/6 mice. Institutional committees approved the acquisition and isolation of human and mouse erythrocytes.

### Erythrocyte hemolysis

Cytotoxicity of ZIF-67 was analyzed in murine and human erythrocytes as described previously [30]. Briefly, 500 μl of blood was gently mixed with 4.5 ml of PBS and 10 μl heparin (1000 u ml^−1^) at 4 °C. This mix was centrifuged at 800 × *g* for 10 min to obtain the cell pellet, the supernatant was carefully eliminated, and the cells were gently resuspended in 200 μl of PBS. 90 μl of the erythrocyte suspension were mixed with 10 μl of ZIF-67 aqueous solution at 8000, 40,000 or 80,000 μg ml^−1^. 2-methylimidazole and cobalt nitrate hexahydrate were also assessed at equimolar amounts to those for ZIF-67. 10 μl of water or Triton X-100 (1% v/v), instead of ZIF-67 solutions, were used as negative and positive hemolysis controls, respectively. The mixes were incubated at 37 °C for 1 h and then centrifuged at 800 × *g* for 15 min at 4 °C. Hemolysis was quantified by determining the absorbance at 550 nm of the supernatants. Hemolysis percentage was calculated using the following formula: % hemolysis = 100% × [(A − A_0_)/(A_t_ − A_0_)]. Where A represents the absorbance of the sample at 550 nm, A_0_ and A_t_ represent 0% and 100% of hemolysis, obtained with water and Triton X-100, respectively.

### Cytotoxicity on human lung cells

Cell viability was determined as previously reported [31]. Shortly, A549 cells (ATCC CCL-185), a human lung carcinoma cell line, were seeded in a 96-well microplate at a density of 1.5 × 10^4^ cells per well, in 100 µl of DMEM Advanced medium (Gibco®) supplemented with 5% fetal bovine serum, 2 mM glutamine, 100 U of penicillin and 50 μg ml^−1^ streptomycin (Gibco®). The microplates with the cells were incubated for 24 h at 37 °C in an atmosphere with 5% CO_2_. The medium was removed and replaced with fresh medium containing ZIF-67 at 800, 4000 or 8000 μg ml^−1^. H_2_O and DMSO 30%, instead of ZIF-67, were used as negative and positive cytotoxicity controls, respectively. The A549 cells were incubated for additional 3.5 h at 37 °C in an atmosphere with 5% CO_2_. Then, the medium was removed and the A549 cells were carefully washed twice with PBS. The microplate containing the A549 cells was inverted and tapped gently to remove excess liquid. 50 µl of a 0.5% crystal violet staining solution in methanol were added to each well and the microplate was incubated at room temperature for 20 min. The staining solution was removed and the A549 cells were washed with 300 µl of deionized H_2_O. The plate was then inverted and placed on a paper towel to air dry for 16 h. 200 µl of methanol were added to each well and the microplate plate was incubated for 20 min at room temperature to solubilize the crystal violet dye adhered to the cells. Then, absorbance of the samples was determined at 570 nm. A decrease in the absorbance values indicates lower amounts of viable cells that remained attached to the microplate wells.

### Statistical analysis

Statistical significance for our experimental results was determined by Student’s *t* test, using GraphPad Prism v8.0.1 software (GraphPad Inc., San Diego, CA.). A *p* ≤ 0.05 was considered significant.

## Results

### X-Ray diffraction of synthesized ZIF-67 nanoparticles

**Fig. 1:**
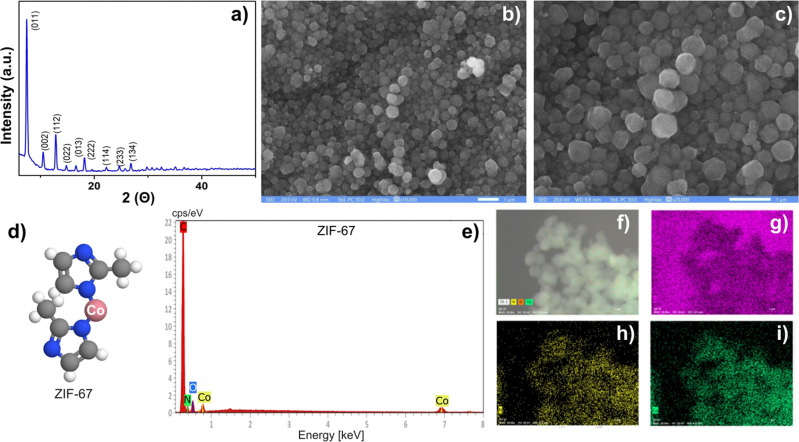
Analysis of synthesized ZIF-67 nanoparticles. **a** X-ray diffraction pattern of ZIF-67, **b**, **c** scanning electron micrographs at different magnifications of ZIF-67 nanoparticles, **d** model for ZIF-67 molecule. Color-coded atoms: blue, nitrogen; gray, carbon; white, hydrogen; pink, cobalt, **e** energy-dispersive spectra of ZIF-67 nanoparticles, and elemental mapping of ZIF-67 nanoparticles: **f** carbon, **g** oxygen, **h** nitrogen and, **i** cobalt

To determine the structural properties of the synthesized ZIF-67 nanoparticles, their X-ray diffraction was recorded. The obtained signals were in agreement with previous reports [32, 33]; peaks corresponding to the crystal planes of ZIF-67 (011, 002, 112, 022, 222, 114, 233, and 134) were observed (Fig. 1a).

### Microscopy analysis of synthesized ZIF-67 nanoparticles

Morphological analysis of the ZIF-67 nanoparticles was carried out by scanning electron microscopy. As shown in Figs. 1b, c, at different magnifications, particles with a uniform dodecahedral morphology and sizes ranging from 100 to 400 nm were clearly visible from the scanning electron micrographs. Figure 1d shows a model of ZIF-67 with a Co bridging two imidazolate residues. Energy-dispersive spectra (EDS) (Fig. 1e) and elemental mapping (Fig. 1f–i) showed the presence of C, N, Co, and O elements, indicating that the ZIF-67 sample is pure and free from contamination.

### ZIF-67 nanoparticles show anti-bacterial activity

**Fig. 2:**
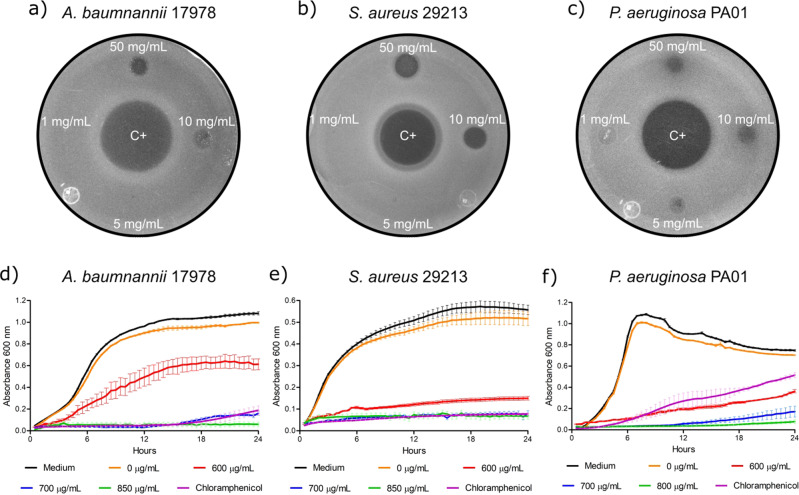
Growth inhibitory effect of ZIF-67 against pathogenic bacteria. ZIF-67 was tested at 50, 10, 5 and 1 mg ml^−1^ on bacteria spread on MH agar: **a** *A. baumannii* 17978, **b** *P. aeruginosa* PA01, and **c** *S. aureus* 29213. The formation of inhibition halos was observed after 24 h of incubation at 37 °C. Gentamycin at 25 μg ml^−1^ was used as a positive control (C+) for the formation of growth inhibition halos. Growth kinetics were obtained for: **d** *A. baumannii* 17978, **e** *S. aureus* 29213, and **f** *P. aeruginosa* PA01, in MH broth containing or not different concentrations of ZIF-67. Chloramphenicol at 12 μg ml^−1^ was used as positive control for growth inhibition. Only medium or medium added with water (solvent for ZIF-67; 0 μg ml^−1^) were assessed as the conditions favorable for growth. Lines represent the average of triplicates; error bars represent SEM

Antibacterial activity of the ZIF-67 nanoparticles was tested against pathogenic bacteria. First, different concentrations of ZIF-67 in aqueous solution (1, 5, 10, and 50 mg ml^−1^) were directly dropped on pathogenic bacteria spreads on MH agar plates and the apparition of an inhibition halo was monitored. Interestingly, at concentrations of 10 and 50 mg ml^−1^ ZIF-67 formed visible inhibition halos on the Gram-negative bacteria *Acinetobacter baumannii* 17978 and *Pseudomonas aeruginosa* PA01, as well as on the Gram-positive bacteria *Staphylococcus aureus* 29213 (Fig. 2a–c). As expected, the antibiotic gentamycin, used as a positive control, also formed inhibition halos on the bacteria tested (Fig. 2a–c).

We then determined the antibacterial effect of ZIF-67 in liquid medium, by obtaining growth kinetics for the bacteria tested in the presence of different concentrations of ZIF-67. In agreement with the results obtained on solid medium, the ZIF-67 nanoparticles inhibited the growth of the *A. baumannii* 17978, *P. aeruginosa* PA01, and *S. aureus* 29213 strains, from a concentration of 700 μg ml^−1^; as expected, the antibiotic chloramphenicol, used as a positive control, also inhibited the growth of the tested bacteria (Fig. 2d–f).

**Fig. 3:**
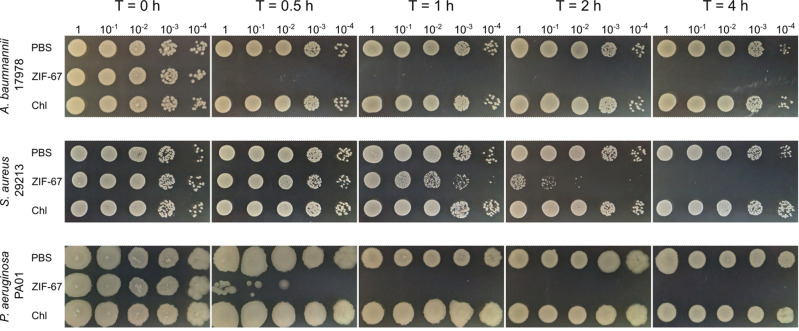
Kill rates of ZIF-67 against pathogenic bacteria. Bacterial suspensions of *A. baumannii* 17978, *P. aeruginosa* PA01, and *S. aureus* 29213 were exposed to ZIF-67 at 850 μg ml^−1^, chloramphenicol (Chl) at 12 μg ml^−1^ or PBS for 4 h at 37 °C. Cell viability was determined by spotting samples of serial 1:10 dilutions of the bacterial suspensions on MH agar plates at different exposition times

Finally, we evaluated the effect of ZIF-67 on cell viability through time of exposition. Bacterial suspensions of *A. baumannii* 17978, *P. aeruginosa* PA01, and *S. aureus* 29213 were exposed to ZIF-67 (850 μg ml^−1^) or chloramphenicol (12 μg ml^−1^), which is a bacteriostatic antibiotic. Then, cell viability was observed by spotting samples of serial 1:10 dilutions of the bacterial suspensions on MH agar plates at different exposition times. Interestingly, ZIF-67 completely killed *A. baumannii*, *P. aeruginosa*, and *S. aureus* cells after 0.5, 1, and 4 h of exposition, respectively (Fig. 3), which indicates a bactericidal effect of ZIF-67. As expected, chloramphenicol did not affect cell viability (Fig. 3); but it inhibited the growth of the bacteria tested (Fig. 2d–f).

In all, these results show that the ZIF-67 nanoparticles have growth-inhibitory and bactericidal activities against pathogenic bacterial species.

### ZIF-67 nanoparticles induce eradication of bacterial biofilms

**Fig. 4:**
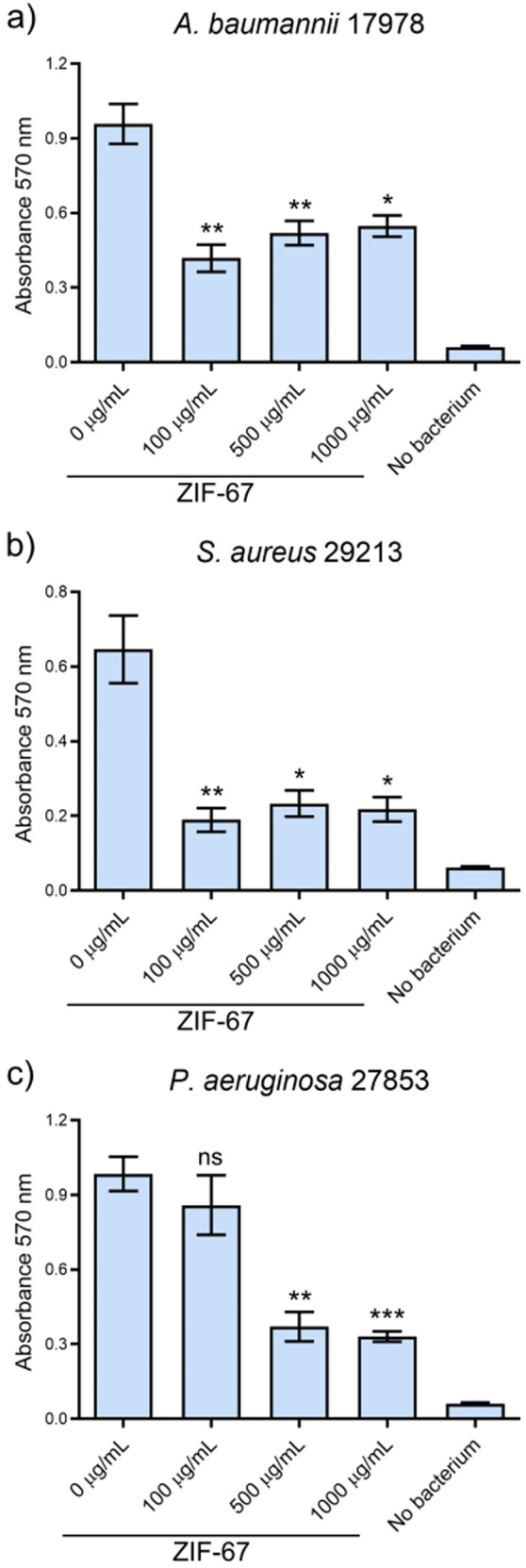
Antibiofilm effect of ZIF-67. Preformed biofilms of **a** *A. baumannii* 17978, **b** *S. aureus* 29213, and **c** *P. aeruginosa* 27853, were treated with different concentrations of ZIF-67 for 24 h and then biofilm biomass retained in the wells was quantified by crystal violet staining. Columns represent the averages of triplicates; error bars represent SEM. Data statistically different with respect to that obtained in the absence of ZIF-67 (0 μg ml^−1^) are indicated: ns not significant; *P ≤ 0.05; **P ≤ 0.009; ***P ≤ 0.0009

Biofilm eradication is a major challenge to combat infection of bacteria such as *A. baumannii*, *P. aeruginosa*, and *S. aureus*. We were interested in investigating whether the ZIF-67 nanoparticles can affect pre-formed biofilms of these bacteria on plastic surfaces. We allowed bacteria to establish biofilms in a 96-well microplate for 24 h; then, the planktonic cells were removed and the pre-formed biofilms were exposed to different concentrations of ZIF-67; after 24 h the biofilm biomass that remained attached to the wells was quantified by crystal violet staining. Notably, the ZIF-67 nanoparticles induced eradication of biofilm formed by *A. baumannii* 17978, *P. aeruginosa* 27853 (a better biofilm former strain than *P. aeruginosa* PA01 in the conditions tested), and *S. aureus* 29213, even at concentrations (100 or 500 μg ml^−1^) below of the concentration (700 μg ml^−1^) that inhibited the growth of these bacteria (Fig. 4). For *A. baumannii* 17978 and *S. aureus* 29213 biofilms we observed a ~ 65 % reduction in the biofilm that remained attached to the wells, as compared to the control without ZIF-67. This was the case even with the lowest tested ZIF-67 concentration (100 μg ml^−1^). For *P. aeruginosa* 27853 we observed a reduction of biofilm biomass only at ZIF-67 concentrations higher than 500 μg ml^−1^. We analyzed the ability to eradicate pre-formed biofilms of various antibiotics commonly used against infections caused by the bacteria in study. The antibiotics were tested at concentrations defined as inhibitory against the examined species by the Clinical and Laboratory Standards Institute [34]. None of the tested antibiotics demonstrated a higher antibiofilm activity than that of ZIF-67 under our experimental conditions (Supplementary Fig. S1). Together, our results indicate that the ZIF-67 nanoparticles can significantly reduce established biofilms.

### Toxicity analysis on mammalian cells for the ZIF-67 nanoparticles

Having shown that the ZIF-67 nanoparticles have antibacterial and antibiofilm activities, we next aimed to determine whether they are harmful towards eukaryotic cells.

**Fig. 5:**
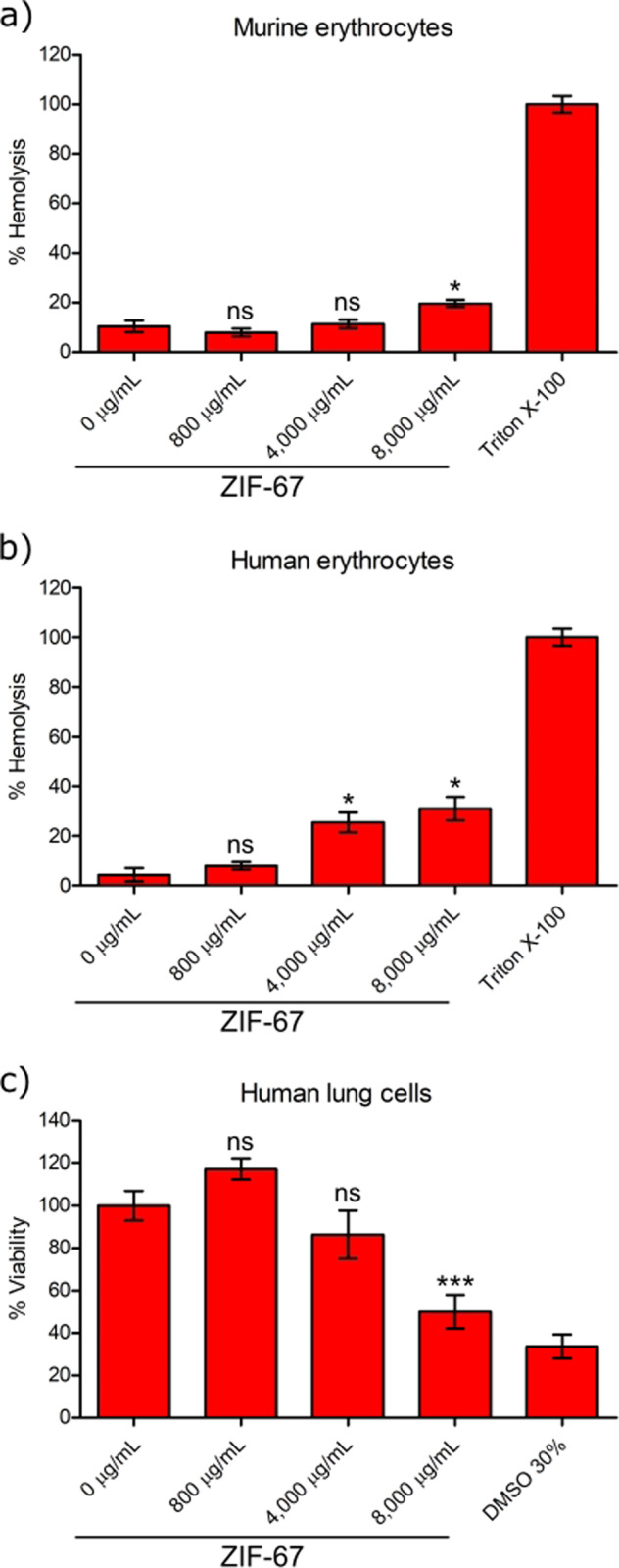
Analysis of hemolytic and cytotoxic activities of ZIF-67 nanoparticles. **a** Murine and **b** human erythrocytes were incubated for 1 h with different concentrations of ZIF-67 and hemolytic activity was then assessed by quantifying the release of hemoglobin. 1% Triton X-100 was used as a positive control for hemolysis. **c** Human lung carcinoma A549 cells were incubated for 3.5 h with different concentrations of ZIF-67, cell viability was then determined by crystal-violet staining. 30% DMSO was used as a positive control for cytotoxicity. Columns represent the averages of triplicates; error bars represent SEM. Data statistically different with respect to that obtained in the absence of ZIF-67 (0 μg ml^−1^) are indicated: ns, not significant; *P ≤ 0.05; ***P ≤ 0.0009

Firstly, assays to evaluate hemolysis activity on human and mice erythrocytes were performed. At a concentration of 800 μg ml^−1^ ZIF-67 did not cause hemolysis of the erythrocytes tested (Fig. 5a, b). Important to note, this is a concentration higher than those of ZIF-67 showing antibacterial or antibiofilm activities (100, 500 or 700 μg ml^−1^). At concentrations 5 and 10 times higher (4000 and 8000 μg ml^−1^, respectively) ZIF-67 caused significant hemolysis in human or in both human and murine erythrocytes; however, this hemolytic effect was still low compared with that produced by Triton X-100 used as a positive control for hemolysis (Fig. 5a, b).

Finally, we examined whether ZIF-67 affects viability of the lung carcinoma cell line A549, which is used as a model of alveolar pulmonary epithelium [35, 36]. Interestingly, at concentrations of 800 and 4000 μg ml^−1^ ZIF-67 did not affect the viability of the tested lung cells, whereas at a concentration of 8000 μg ml^−1^ ZIF-67 caused a ~50% loss of cell viability (Fig. 5c).

Our results support that the ZIF-67 nanoparticles are not harmful for mammalian cells at concentrations where they show antibacterial and antibiofilm effects.

## Discussion

In this study, we synthesized ZIF-67 nanoparticles and found that these inhibit the growth and show bactericidal activity against *A. baumannii*, *P. aeruginosa*, and *S. aureus* (Figs. 1–3). Previously, composite materials that incorporated ZIF-67 into alginate fibers or paper were shown to possess antibacterial activity against *E. coli* [25, 37]. These materials consist of two or more distinct components that combine to form a new material with improved properties. For instance, composite alginate-based antibacterial fibers made up of alginic acid, a polysaccharide, are developed through blend spinning with ZIF-67. These fibers demonstrate high tensile strength and antibacterial properties, making them a promising candidate for high-performance antibacterial textiles [25]. Moreover, a biodegradable paper-based composite material modified with ZIF-67 and composed of carboxylated cellulose nanofibers exhibits robust mechanical performance under varying humidity conditions and considerable bacteriostatic effects [37]. We observed antibacterial effect of ZIF-67 in aqueous solution, against both Gram-negative and Gram-positive pathogenic bacterial species.

Interestingly, supporting a possible use of ZIF-67 as an antibacterial compound, our ZIF-67 nanoparticles did not cause hemolysis in human and murine erythrocytes, nor affected the viability of A549 lung cells, when tested at a concentration effective against bacteria (800 μg ml^−1^). Indeed, even concentrations 5-fold higher (4000 μg ml^−1^) showed no cytotoxic effect against A549 lung cells nor against murine erythrocytes, and only a moderate hemolytic effect against human erythrocytes (Fig. 5). The most notable difference between different ZIF configurations is the transition metal ions incorporated within their structure. ZIF-8 is considered the most well-known type of this MOF family. It is composed of the metal cation Zn^2+^ linked to 2-methylimidazole ligand species, resulting in large cavities of approx. 1.16 nm interconnected through windows of about 0.3 nm, as calculated by X-ray diffraction analysis [38]. ZIF-8 particle size and crystallinity can vary depending on the synthesis conditions, but in general they present high porosity and surface area, which confers them potential as drug delivery systems; although ZIF-8 has been shown to have an intrinsic dose-dependent cytotoxicity [21, 39]. Interestingly, the cytotoxicity of ZIF-8 can be managed by coating its nanoparticles with different materials, which controls its release of Zn and even allows for improved anticancer-drug@ZIF-8 composites by making use of the cytotoxicity of ZIF-8 against targeted tumoral cells [40]. The cytotoxicity of ZIF-67 has not been investigated thoroughly. Sun et al. showed that the toxicity of Co ions released by ZIF-67 was lower than that of CoCl_2_ when both compounds were tested in a hydrogel preparation [41]. More studies are required to better determine the biocompatibility of ZIF-67.

Previous reports indicate that cobalt has antibacterial activity, probably due to its ability to cross bacterial membranes and to exert a strong oxidative effect [42, 43]. Accordingly, in our assays cobalt nitrate hexahydrate inhibited the growth of the bacteria tested, at equimolar concentrations of those of ZIF-67 showing antibacterial activity; 2-methylimidazole (the other substrate for the synthesis of ZIF-67) partially inhibited the growth of only *S. aureus* (Supplementary Fig. S2). Nevertheless, cobalt nitrate hexahydrate showed higher hemolytic activity than ZIF-67 (Supplementary Fig. S3). Therefore, it is reasonable to think that ZIF-67 favors cobalt-mediated antibacterial activity with less toxic effect for mammalians cells than cobalt ions.

We found that the ZIF-67 nanoparticles also have antibiofilm activity, even higher than some antibiotics tested; they removed bacterial biofilm attached to a plastic surface (Fig. 4). Interestingly, the ZIF-67 nanoparticles eradicated preformed biofilms of *A. baumannii*, *P. aeruginosa*, and *S. aureus* strains at concentrations of 100 or 500 μg ml^−1^, which are concentrations below that showing antibacterial effect (700 μg ml^−1^). These results suggest that the antibacterial and antibiofilm activities of ZIF-67 are independent. Recently, ZIF-8 and ZIF-L have been reported to possess good dose-dependent antibiofilm activity against pathogenic bacteria such as methicillin-resistant *S. aureus, P. aeruginosa* and *Porphyromonas gingivalis*, either by themselves or in combination with other compounds [44–47]. The acidic pH commonly found in bacterial biofilms favors the disintegration of ZIFs frameworks, causing an abundant release of metallic ions which can penetrate the biofilm and exert their antibiofilm effect [44]. This mechanism of metal-ion release is similar to ones previously reported for different pH-sensitive MOFs [13, 16]. In 2023, Ge et al. reported that composite nanoparticles of Na_2_S_2_O_8_@ZIF-67 with a glucose oxide coadjuvant were able to efficiently eradicate mixed biofilms of *S. aureus* and *Candida albicans*; this antibiofilm effect was due to the generation of reactive oxygen species by Na_2_S_2_O_8_ after it was released from a ZIF-67 carrier by low pH [48].

To our knowledge, our work presents the first report of ZIF-67 as a bona fide antibiofilm agent, showing that it can eradicate preformed bacterial biofilms by itself. Our findings, together with previous reports [21, 49], support that ZIF-67 nanoparticles could have potentially broad applications as antibacterial compounds. However, further investigations are required to determine whether this MOF can be used, alone or in combination with other compounds, to combat bacterial infections and/or eradicate bacterial biofilms in vivo.

### Supplementary information

Supplementary information
