# Supplementary material for: Antibacterial and antibiofilm activities of ZIF-67
Source: J Antibiot (Tokyo). 2023 Jun 19;76(10):603–12. doi: 10.1038/s41429-023-00637-8 (PMC10522484; doi:10.1038/s41429-023-00637-8)
Supplement: Supplementary file 1 — Supplementary information [file 41429_2023_637_MOESM1_ESM.docx]

**Supplementary information**

**Antibacterial and antibiofilm activities of ZIF-67**

Ramses Gallegos-Monterrosa^a^, Rodrigo Orozco Mendiola^b^, Yoselin Nuñez^c^, Constance Auvynet^c^, Kesarla Mohan Kumar ^b^, Bin Tang^d^, Leonardo I. Ruiz-Ortega^b,e,#^ and Víctor H. Bustamante^a,§^.

^a^ Departamento de Microbiología Molecular, Instituto de Biotecnología, Universidad Nacional Autónoma de México, C.P. 62210, Cuernavaca, Morelos, México.

^b^ Instituto de Ciencias Físicas, Universidad Nacional Autónoma de México, C.P. 62210, Cuernavaca, Morelos, México.

^c^ Departamento de Medicina Molecular y Bioprocesos, Instituto de Biotecnología, Universidad Nacional Autónoma de México, C.P. 62210, Cuernavaca, Morelos, México.

^d^ Department of Biomedical Engineering, Southern University of Science and Technology, 1088 Xueyuan Avenue, Shenzhen 518055, P.R. China.

^e^ Department of Biological Sciences, Columbia University, New York, 10027, USA.

^#^ Corresponding author at: +52 6623737418, lr3113@columbia.edu

^§^ Corresponding author at: +52 7773291627, victor.bustamante@ibt.unam.mx

**Fig. S1**


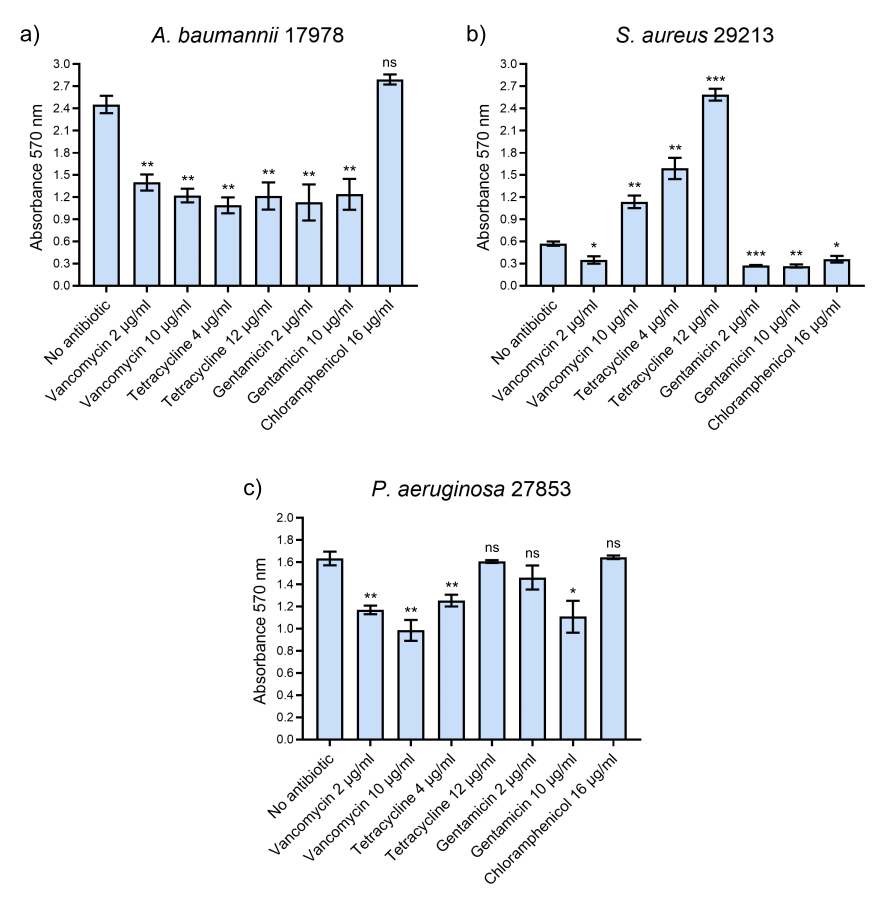


**Figure S1:** Antibiofilm effect of antibiotics vancomycin, tetracycline, gentamicin, and chloramphenicol. Preformed biofilms of **a)** *A. baumannii* 17978, **b)** *S. aureus* 29213, and **c)** *P. aeruginosa* 27853, were treated or not with the indicated concentrations of antibiotics for 24 h; then the biofilm biomass retained in the wells was quantified by crystal violet staining. Columns represent the averages of triplicates; error bars represent SEM. Data statistically different with respect to that obtained in the absence of antibiotic are indicated: ns, not significant; *, *P* ≤ 0.05; **, *P* ≤ 0.009; ***, *P* ≤ 0.0009.

**Fig. S2**


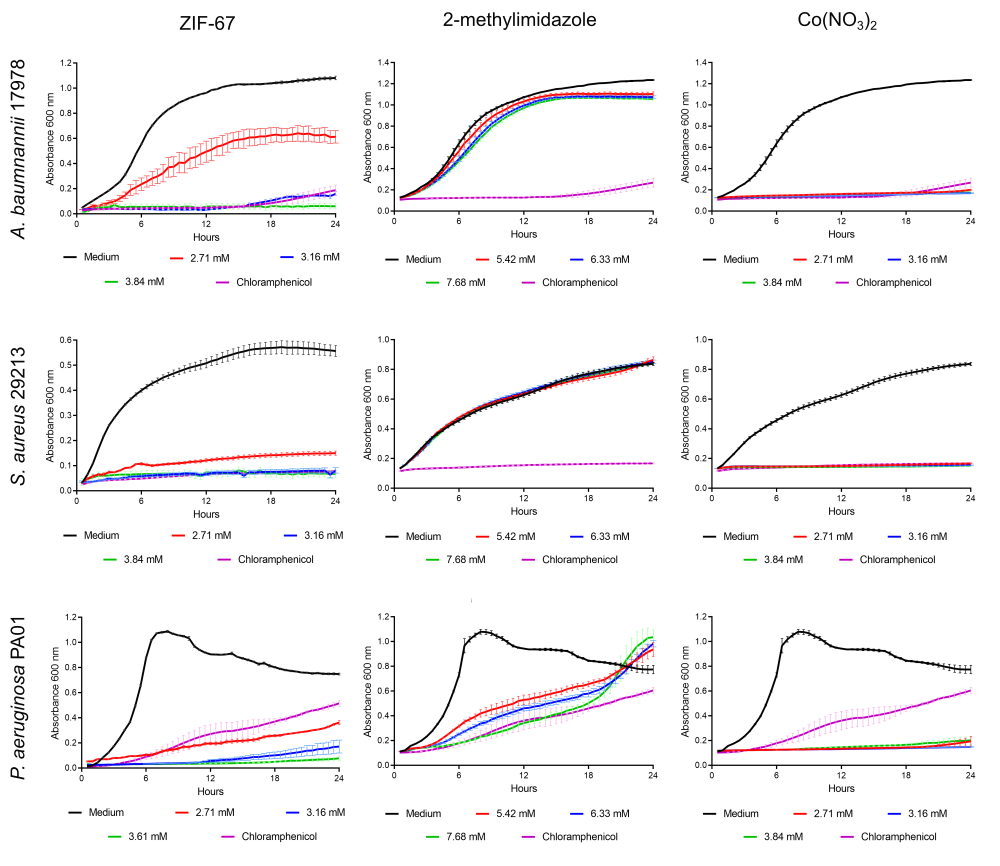


**Figure S2:** Growth inhibitory effect of ZIF-67, 2-methylimidazole, and cobalt nitrate hexahydrate against pathogenic bacteria. Growth kinetics were obtained for *A. baumannii* 17978, *S. aureus* 29213, and *P. aeruginosa* PA01 in MH broth containing the indicated concentrations of ZIF-67, 2-methylimidazole (2-MI) or cobalt nitrate hexahydrate (Co(NO_3_)_2_). Chloramphenicol at 12 μg/mL was used as positive control for growth inhibition. Medium without any other compound was assessed as the condition favorable for growth. Concentrations of ZIF-67 are the same used for Figs. 2d-f but shown as molarity for a better comparison. Lines represent the average of triplicates; error bars represent SEM.

**Fig. S3**


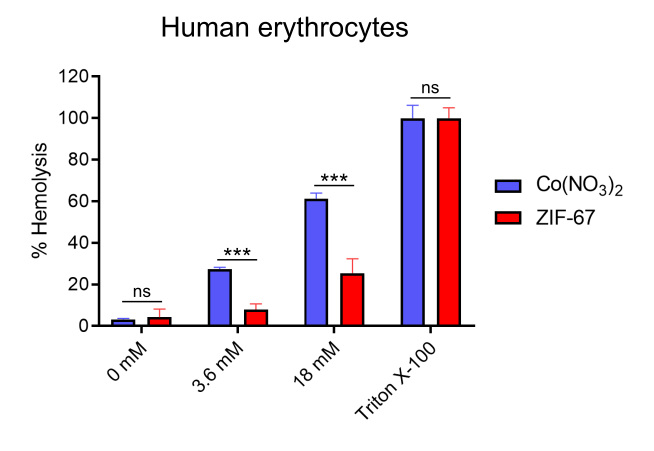


**Figure S3:** Analysis of the hemolytic activity of ZIF-67 and cobalt nitrate hexahydrate. Human erythrocytes were incubated for 1 h with the indicated concentrations of ZIF-67 or cobalt nitrate hexahydrate (Co(NO_3_)_2_); then, hemolytic activity was assessed by quantifying the release of hemoglobin. 1% Triton X-100 was used as a positive control for hemolysis. Concentrations of ZIF-67 are the same used for Fig. 5b but shown as molarity for a better comparison. Columns represent the averages of triplicates; error bars represent SEM. Horizontal bars indicate statistical differences between treatment comparisons: ns, not significant; ***, *P* ≤ 0.0009.
